# Production and structural characterization of a new type of polysaccharide from nitrogen-limited *Arthrospira platensis* cultivated in outdoor industrial-scale open raceway ponds

**DOI:** 10.1186/s13068-019-1470-3

**Published:** 2019-05-24

**Authors:** Qishun Liu, Changhong Yao, Yongxin Sun, Wei Chen, Haidong Tan, Xupeng Cao, Song Xue, Heng Yin

**Affiliations:** 1 Division of Biotechnology, Dalian Institute of Chemical Physics, Chinese Academy of Sciences, Dalian, 116023 China; 2 Department of Pharmaceutical & Biological Engineering, School of Chemical Engineering, Sichuan University, Chengdu, 610065 Sichuan China; 3 Dalian Biotechnology Research Institute, Liaoning Academy of Agricultural Sciences, Dalian, 116024 China; 4 Liaoning Provincial Key Laboratory of Carbohydrates; Dalian Engineering Research Center for Carbohydrate Agricultural Preparations, Dalian Institute of Chemical Physics, Chinese Academy of Sciences, Dalian, 116023 China

**Keywords:** *Arthrospira platensis*, Carbohydrate, Nitrogen limitation, Purification

## Abstract

**Background:**

Carbohydrates are major biomass source in fuel-targeted biorefinery. *Arthrospira platensis* is the largest commercialized microalgae with good environmental tolerance and high biomass production. However, the traditional target of *A. platensis* cultivation is the protein, which is the downstream product of carbohydrates. Aiming to provide the alternative non-food carbohydrates source, the feasible manipulation technology on the cultivation is needed, as well as new separation methodology to achieve maximum utilization of overall biomass.

**Results:**

The present study aimed to demonstrate the feasibility of industrially producing carbohydrate-enriched *A. platensis* and characterize the structure of the polysaccharide involved. Cultivated in industrial-scale outdoor open raceway ponds under nitrogen limitation, *A. platensis* accumulated maximally 64.3%DW of carbohydrate. The maximum biomass and carbohydrate productivity reached 27.5 g m^−2^ day^−1^ and 26.2 g m^−2^ day^−1^, respectively. The efficient extraction and purification of the polysaccharides include a high-pressure homogenization-assisted hot water extraction followed by flocculation with a non-toxic flocculant ZTC1 + 1, with the polysaccharide purity and total recovery reaching 81% and 75%, respectively. The purified polysaccharide was mainly composed of (1→3)(1→4)- or (1→3)(1→2)-α-glucan with a molecular weight of 300–700 kDa, which differed from the commonly acknowledged glycogen.

**Conclusions:**

By the way of controlled nitrogen limitation, the high carbohydrate production of *A. platensis* in the industrial scale was achieved. The α-glucan from *A. platensis* could be a potential glucose source for industrial applications. A non-toxic separation method of carbohydrate was applied to maintain the possibility of utilization of residue in high-value field.

**Electronic supplementary material:**

The online version of this article (10.1186/s13068-019-1470-3) contains supplementary material, which is available to authorized users.

## Background

Microalgae, which can efficiently covert solar energy and carbon dioxide into biomass, is considered as the third generation of biomass sources following the first generation (crops such as corn, sugar cane, oil crops, etc.) and the second generation (lignocellulose such as straw, forestry waste, etc.) feedstock for biorefinery [1, 2]. The major advantage of using microalgae is their high photosynthetic efficiency (10–50 times higher than higher plants) with the potentiality of continuous and whole-year cultivation with little need for arable land or potable water [3, 4]. Carbohydrate, especially storage polyglucan such as glycogen in cyanobacteria and starch in eukaryotic algae, has been regarded as sustainable carbon source for microbial fermentation in the production of bioenergy (e.g., bioethanol and biobutanol) and other glucose-based industry [3, 5–7]. In addition, polysaccharides from microalgae are also considered to be high-value compounds that can be applied in food, clinical drugs, cosmetics, and other chemical industry [8]. Therefore, production of microalgal storage carbohydrate and functional polysaccharides has attracted increasingly attention [8–10].

*Arthrospira* (*Spirulina*), a filamentous cyanobacterium with various applications and considerable markets, is the largest commercially produced microalga in China and in the world [11]. Industrial production of *Arthrospira* (*Spirulina*) biomass is usually achieved in outdoor open raceway ponds due to its favorable properties such as alkaline cultivation conditions (pH 9 to 11) which minimizes contamination. It is famous for its unusual high amounts of balanced proteins (> 60%DW) which is useful in the health products and aquaculture, therefore nitrogen repletion is generally required in large-scale cultivation of *Arthrospira* (*Spirulina*) [12]. Conversely, nitrogen deprivation has been shown to trigger protein turnover with the accumulation of carbohydrate up to more than 70%DW in *A. platensis* at a lab scale [13–15]. Carbohydrate-enriched *A. platensis* has been demonstrated to be a promising feedstock for bioethanol production under a biorefinery approach [16]. The fermentation requires large amounts of carbohydrate-enriched algal biomass which has to be produced industrially. However, to the best our knowledge, there is scarce report regarding the microalgal carbohydrate production at an industrial scale under natural outdoor conditions.

Generally, glycogen, a highly branched (1→4)(1→6)-linked α-glucan, is considered to be the storage carbohydrate in cyanobacteria under nitrogen starvation [3, 17, 18]. Although currently it is accepted that glycogen is the storage polyglucan in *A. platensis* under nutrient starvation [3, 13, 14], the conclusion is in fact only deduced via an extrapolation from the phylum (cyanobacteria) that *A. platensis* belongs to. There is very limited information regarding to the structure, especially the glycosidic linkage, of the storage carbohydrate in this alga, which needs in-depth characterization.

For structural characterization and downstream applications, it is essential to extract and purify the polysaccharides from algae. Several methods have been used to extract the water-soluble polysaccharides in *A. platensis*, such as hot water [19] or hot base [17] extraction, organic solvents extraction [20], and ultrasound [21] or microwave [22] assisted hot water extraction. For polysaccharide purification, Sevage refining, trichloroacetic acid (TCA) precipitation, enzymolysis, or the combination of these methods can be applied to remove the protein and other impurities [23, 24]. However, these traditional methods often introduce organic solvents and are also labor-intensive [23]. Recently, chitosan flocculation along with macroporous adsorption was developed to efficiently improve polysaccharide purity with high recovery rate [25], which represented a greener way for polysaccharide purification. However, these methods were used in the nitrogen-replete *A. platensis*. The development of extraction and purification methods for polysaccharides from nitrogen-limited *A. platensis* is still required.

To demonstrate the feasibility of industrially producing carbohydrate-enriched *A. platensis* biomass and characterize the structure of the water-soluble polysaccharides contained in it, the present study used nitrogen limitation strategy for the cultivation of *A. platensis* in outdoor industrial-scale open raceway ponds. The efficient and green approach for extraction and purification of the polysaccharides from these kinds of alga was then developed by comparing different methods. Finally, the structure of the purified polysaccharide was elucidated via chemical and instrumental analysis.

## Methods

### Algal strain and cultivation conditions

**Fig. 1:**
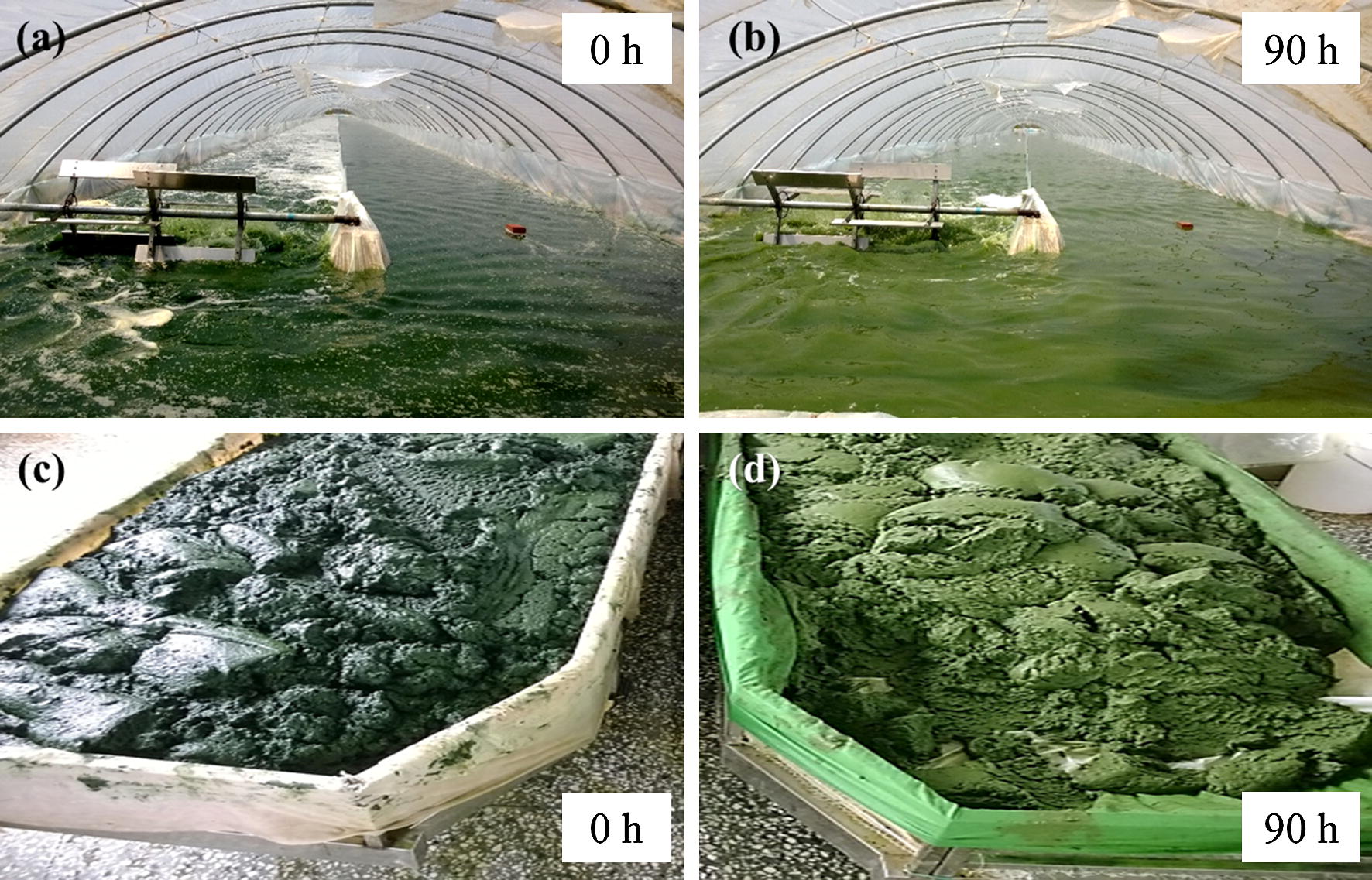
Cultivation and harvest of *A. platensis* in industrial-scale open raceway ponds under nitrogen-limited conditions. The normal algae paste with dark green (**c**), were inoculated in the raceway pond (**a**) and subjected to nitrogen-limited conditions for 90 h (**b**), before harvesting to yield light green algal paste (**d**)

Algal paste obtained from the commercially produced *A. platensis* in Ordos Lvfuyuan Biotechnology Corp. Ltd. (China) was used as the seed for the production of polysaccharide-enriched algal biomass in the same company. The algae were cultivated in two independent raceway ponds with a length of 110 m, width of 6 m, and depth of 0.3 m that have a working volume of approximately 180 m^3^. The cultures were driven by paddlewheels which generated a flow velocity of about 0.2 m s^−1^ (Fig. 1). This cultivation system was originally applied for industrial production of *A. platensis* biomass by Ordos Lvfuyuan Biotechnology Corp. Ltd. (Ordos, China).

The algal paste (10% dry biomass) harvested and washed during the industrial production was inoculated into fresh nitrogen-free medium so that the initial cell density was approximately 0.26 g/L. The medium consisted of (per m^3^): natural sodium bicarbonate (from the nearby alkaline lakes) 6 kg, KCl 0.5 kg, MgSO_4_·7H_2_O 0.01 kg, FeSO_4_·7H_2_O 0.01 kg, and H_3_PO_4_ (85% w/w) 0.12 kg. The natural sodium bicarbonate was obtained by sun drying of the alkaline lake water. This crude natural soda which contained more than 50% (w/w) of sodium bicarbonate was partially applied in the industrial cultivation of *A. platensis* in Ordos [26]. The cultures were subjected to natural sunlight and the temperature varied from 25 to 35 °C during the diurnal cycles (Additional file 1: Fig. S1). The algal biomass (dry weight, DW, g L^−1^), nitrate concentration (NO_3_^−^-N, mg/L) in the medium, carbohydrate or protein content (%DW) in the biomass as well as the biomass and carbohydrate productivities were tracked during the cultivation. The methods for the measurement of these parameters were described as Yao et al. [15]. The cultivation started at 15:30, Jul 4th 2014, and lasted for 90 h before the algal cells were harvest by filtration, washed with ground water, and finally subjected to spray drying.

### Extraction and purification of water-soluble polysaccharides from *A. platensis*

#### Extraction of polysaccharides

Three extraction methods were applied to make a comparison in terms of polysaccharide extraction efficiency. (1) Hot water extraction of polysaccharide: 10 g of algae powder was added to 0.3 L distilled water (1:30, w/v), stirred at 150 rpm for 4 h at 80 °C, and then centrifuged at 6000 rpm for 20 min to remove the debris, and the supernatant was obtained as the crude extract; (2) ultrasonic disruption coupled with hot water extraction of polysaccharide: algal slurry with solid–liquid ratios (w/v) of 1:10, 1:20 and 1:30, respectively, were prepared as described in the hot water extraction method, and the mixtures were placed in an ice-water bath before sonication for three times in an ultrasonic crusher (KS-250, Ningbo Kesheng Instrument Factory) under 500 W for 10 min each time. After disruption, the crude extracts were further subjected to hot water extraction; (3) high-pressure homogenization coupled with hot water extraction of polysaccharide: algal slurry with a solid–liquid ratio of 1:30 were prepared as described in the hot water extraction method, and the mixture was then homogenized at high-pressure homogenizer (AH150, ATS Engineering Inc) at 80 MPa pressure for three times with a flow rate of 100 mL/min. After disruption, the crude extract was further subjected to hot water extraction.

The polysaccharides extraction efficiency (PEE, %) was calculated as follows:

1 $${\text{PEE}} = W_{0} /W_{s} \times 100\%$$

where *W*_*0*_ (g) was the amount of the extracted polysaccharide, and *W*_*s*_ (g) was the amount of the total sugar contained in the dried powder of *A. platensis*. The concentration of total sugar was determined by the phenol–sulfuric acid method [27].

#### Purification of polysaccharides

Different flocculation methods were used to remove the protein and other impurities. Flocculants including ZTC 1 + 1 natural clarifying agent (Beijing Zheng Tiancheng clarification Technology Co., Ltd), chitosan, polyferric sulfate and cationic polyacrylamide were slowly added into the crude extracts, stirring for 30 min before standing for 30 min. The mixture was then centrifuged at 8000 rpm for 20 min to remove the precipitate. For the ZTC 1 + 1 method, the flocculated polysaccharide was further refined by Sevage method [23].

The polysaccharide purity (PP, %) was calculated as follows:

2 $${\text{PP}} = W_{1} /W \times 100\%$$

where *W*_*1*_ (g) was the amount of sugar in the sample, and *W* (g) was the weight of the sample.

The polysaccharide retention rate (PRR, %) was calculated as follows:

3 $${\text{PRR}} = W_{2} /W_{c} \times 100\%$$

*W*_*2*_ (g) was the amount of the sugar in the sample after each treatment (flocculation), and *W*_*c*_ (g) was the amount of the sugar in the sample of the crude polysaccharide extract.

The total polysaccharide recovery rate (TPRR, %) was calculated as follows:

4 $${\text{TPRR}} = {\text{PEE}} \times {\text{PRR}} \times 100$$

After flocculation or refining, fivefold volume of anhydrous ethanol was added to the obtained polysaccharide solution and let stand for 30 min before being subjected to suction filtration. The filter cake was washed for three times with anhydrous ethanol and then freeze-dried to obtain the polysaccharides.

### Characterization of water-soluble polysaccharide from *A. platensis*

#### Molecular weight analysis

The molecular weight of the polysaccharide sample was determined on a gel permeation chromatography (GPC) system where TSK gel G5000 PWxl + G3000 PWxl column (7.8 mm × 300 mm, 10 μm particle diameter, Tosoh Corporation, Tokyo, Japan) was fitted with a Waters 515 HPLC Pump and a Waters 2414 RI detector (Waters Co., Milford, MA, USA). The samples were eluted with 0.01 M NaNO_3_ at pH 7.0 with a flow rate of 0.7 mL/min at 30.0 ± 0.1 °C. The linear regression was calibrated by Pullulan (shodex P-82) as a standard. All data provided by the GPC system were collected and analyzed with the Empower Workstation software package (Waters Corp., MA), following the method described by Alsop et al. [28].

#### The IR spectra of *A. platensis* polysaccharides

The IR spectra of *A. platensis* polysaccharides were determined using a Fourier transform IR (FTIR) spectrophotometer (Bruker Vector 22, USA). The purified polysaccharides were grounded with KBr powder and then pressed into pellets for FTIR measurements in the frequency range of 4000–500 cm^−1^.

#### The ^1^H NMR and ^13^C NMR of *A. platensis* polysaccharides

The ^1^H NMR and ^13^C NMR experiments were recorded at 400 MHz on a Bruker AVANCE III spectrometer using deuterated water as the solvent at 27 °C. Tetramethylsilane (TMS) was used as an internal standard.

#### Sodium periodate oxidation analysis of *A. platensis* polysaccharides

25 mg of the polysaccharide was dissolved in 12.5 mL of distilled water, and 12.5 mL of 30 mmol/L NaIO_4_ were added. The solution was kept in the dark at room temperature. For sampling, aliquots of 0.1 mL were withdrawn at 3–6-h intervals, diluted to 25 mL with distilled water, and measured on a spectrophotometer at 223 nm. Glycol (2 mL) was then added to stop the periodate oxidation reaction. The resulting formic acid was titrated with 0.01 mol/L NaOH solution. The linkage mode of glycoside bonds of the *A. platensis* polysaccharides were calculated according to the consumption of sodium periodate and the formation of formic acid [29].

#### Monosaccharide composition analysis

The polysaccharide (3 mg) was hydrolyzed with 2 mL of 2 M trifluoroacetic acid at 121 °C for 2 h, and the acid was removed by co-distillation with water. The monosaccharides were reduced by NaBH_4_, followed by acidification with acetic acid to alditols. The alditols were then converted into alditol acetates by heating with pyridine-Ac_2_O (v/v 1:1) for 30 min at 100 °C and analyzed by Shimadzu GC-2010 plus (Japan) gas chromatograph equipped with a flame-ionization detector. The instrument was fitted with a ov-17 column (30.0 m × 0.25 mm × 0.25 μm, the Chinese Academy of Sciences Dalian Institute of Chemical Physics). The operation was performed in the following conditions: carrier gas, nitrogen/air; column flow rate, 0.88 mL/min; purge flow, 3.0 mL/min; pressure, 110 kPa; injection volume, 1.0 μL; air flow, 400 mL/min; tail gas flow, 30 mL/min; total flow, 20.6 mL/min; H_2_ flow, 40 mL/min; linear velocity, 28.4 cm/s; detector, 270° C; split ratio, 19:1; programmed temperature, 180 °C (2 min)–6 °C/min–210 °C–0.3 °C/min–215 °C–6 °C/min–240 °C (30 min). Quantitation was carried out from peak areas, using response factors from external standard monosaccharides of d-glucose, d-mannose, l-rhamnose, d-galactose, d-xylose, l-arabinose, d-fucose, d-glucuronic acid, and d-galacturonic acid which were purchased from Sigma Co., Ltd.

## Results and discussion

### Nitrogen-limited culture of *Arthrospira platensis*

**Fig. 2:**
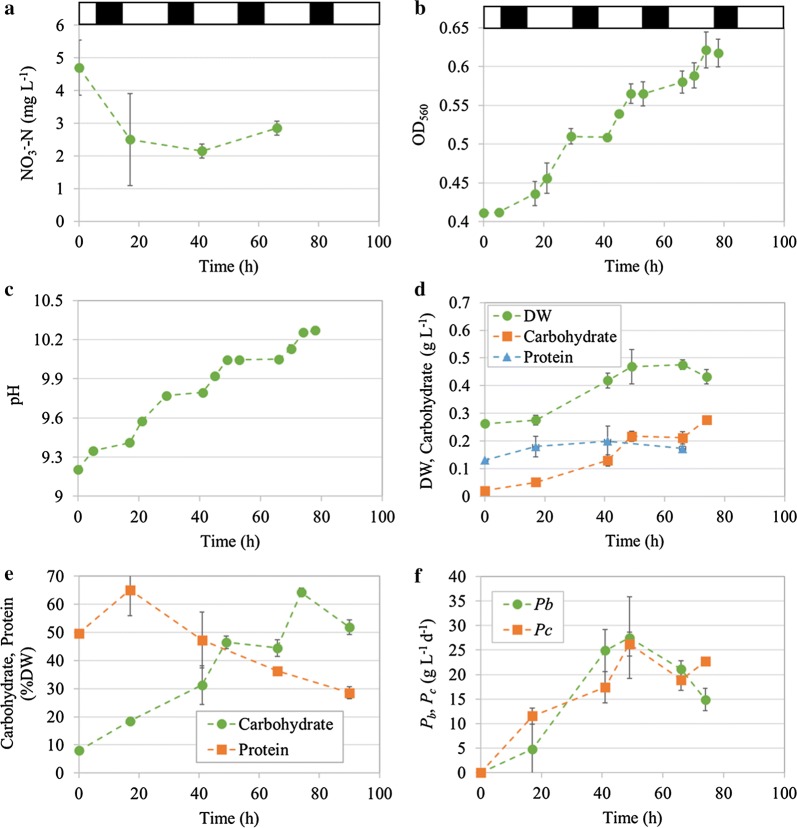
Nitrate consumption (**a**), cell growth (OD_560_, **b**), pH variations (**c**), biomass, carbohydrate or protein concentrations (**d**), carbohydrate or protein content (**e**), and biomass or carbohydrate productivity (**f**) of *Arthrospira platensis* cultivated under nitrogen-limited conditions in industrial-scale raceway ponds. The white and black regions on the top denoted natural light and dark periods of a diurnal cycle, respectively. The data represented the average values of two independent replicated cultivations and the bars showed the standard deviations (mean ± SD)

To demonstrate the feasibility of industrially producing carbohydrate from *A. platensis*, nitrogen-limited cultures were applied in the industrial-scale open raceway ponds. To this end, the algal paste was washed with water to eliminate the extracellular nitrogen source before inoculation. This enabled an initial nitrate concentration of approximately 4.7 mg L^−1^ in the medium (Fig. 2a). Under this N limited condition, *A. platensis* could assimilate very little nitrate (less than 2 mg L^−1^) and proliferate by recycling the nitrogen stored in cells. The OD_560_ denoting the cell growth showed a general increase during the cultivation; after 84 h the cell density increased by 50% compared with the initial level (Fig. 2b). Correspondingly, the pH of the medium also increased from the initial level of 9.2 to finally 10.3. This increase of pH was attributed to the assimilation of bicarbonate, leading to the release of OH^−^ and consequently the alkalization of the medium [30]. This meant that although *A. platensis* was subjected to nitrogen starvation, the photosynthetic carbon fixation continued. As a result, the biomass increased by maximally 80% during 49 h (Fig. 2d). The maximal biomass productivity of 27.5 g m^−2^ day^−1^ was achieved on the third daytime (49 h, Fig. 2f), after which it declined gradually due to the decreased biomass accumulation (Fig. 2d). This could be attributed to the long-term nitrogen stress that affected the photosynthesis and the consequently impaired anabolic metabolism. In fact, the protein content declined from 47.3 to 28.5%DW at the late period of cultivation (41–90 h, Fig. 2e), representing an unfavorable condition that the algae were subjected to. Apparently, the color of the cultures and the harvested algal pastes changed from normal dark green to light green (Fig. 1), which also suggested a degradation of chlorophyll which manifested a declined photosynthesis. On the contrary, as expected, the carbohydrate accumulated under the N-limited conditions (Fig. 2d, e), with the highest carbohydrate content of 64.3%DW obtained at 74 h (Fig. 2e). However, the carbohydrate productivity peaked at 49 h, reaching 26.2 g m^−2^ day^−1^, and thereafter it declined to 22.7 g m^−2^ day^−1^ at 74 h despite the carbohydrate content increased during this period (Fig. 2e, f). Therefore, to balance the discrepancy of carbohydrate content and biomass/carbohydrate productivity, it would be reasonable to harvest the algae on the 3rd day at 49 h when the biomass productivity reached the top level with a carbohydrate content of 46.5%DW.

Noteworthily, it was evident from Fig. 2d, e that the carbohydrate accumulated primarily at daytime (41–49 h, 66–74 h), while at night the accumulation ceased or the carbohydrate even degraded (49–66 h, 74–90 h). Hidasi et al. [31] also found that the carbohydrate content of the *A. platensis* biomass cultivated in large-scale outdoor raceway ponds increased during the light hours, and decreased during the dark hours. Similarly, the variations of cell density and pH exhibited almost a synchronized manner, both of which showed significant increase during the daytime (0–5 h, 17–29 h, 41–53 h, and 66–74 h), while at night remained unchanged (Fig. 2b, c). As discussed above, the increase of pH could be an indicator of photosynthetic carbon fixation and biomass production [30]. Collectively, it indicated that the carbohydrate accumulation could mainly be ascribed to the de novo synthesis from inorganic carbon (bicarbonate) via photosynthesis at daytime. Although protein turnover could be another route to provide carbon skeleton for carbohydrate accumulation in *A. platensis* [13], the contribution of this part should be limited since protein concentration showed little decline herein (Fig. 2d). In view of the significant role of photosynthesis played in biomass and carbohydrate production, it is advisable to harvest the carbohydrate-enriched algae in the late afternoon.

The present study demonstrated that via nitrogen limitation, it is feasible to industrially produce *A. platensis* with more than 45%DW carbohydrate accumulated intracellularly. The carbohydrate content of this level was comparable to most of the microalgae cultivated photoautotrophically in large-scale PBRs (cultivation volume ≥ 0.05 m^3^) under outdoor natural conditions, and it was especially prominent when compared with those cultivated in outdoor raceway ponds (less than 35%, Table 1) [32–34]. In addition, from Table 1, it could also be seen that the maximum biomass productivity of *A. platensis* cultivated under −N conditions in industrial-scale raceway ponds herein (27.5 g m^2^ day^−1^) was comparable to or even exceeded those of *A. platensis* (22–25 g m^2^ day^−1^, [35, 36]) and other microalgae, such as *Scenedesmus acutus* (12.5 g m^2^ day^−1^, [34]) and *Chlorella* sp. L1 (23.1 g m^2^ day^−1^, [37]), cultivated under −N or even +N conditions in the similar type of cultivation system with smaller volumes at similar seasons (summer). In fact, it is rare to get productivities in excess of 25 g m^−2^ day^−1^ in natural large scale systems with current algal cultivation technologies [12]. Therefore, the relatively high biomass productivity obtained under such a large cultivation volume herein should also be attractive. Although the biomass and/or carbohydrate productivity were still inferior to those cultivated in tubular [38] or thin-layer [39] PBRs, the 100–1000-fold larger cultivation volume of the open raceway pond used in the present study should exhibit its advantages in terms of reduced operation costs.

**Table 1:** Comparison of photoautotrophic microalgal biomass and carbohydrate production in large-scale PBRs (cultivation volume ≥ 0.05 m^3^) under outdoor natural conditions

Strain	Cultivation system (volume, m^3^)	Location and season	*P*_ba_ (g m^−2^ day^−1^)	*P*_bv_ (mg L^−1^ day^−1^)	*C*_c_ (%DW)	References
*Monoraphidium dybowskii*	Raceway pond (40)	IM, China, summer	27.8	139.17	NA	[40]
*Chlorella* sp.	Raceway pond (40)	IM, China, summer	23.1	115.35	NA	[37]
*Scenedesmus acutus*	Raceway pond (2.3)	USA, autumn	12.5	NA	32.4	[34]
*Graesiella* sp.	Raceway pond (40)	Yunnan, China, summer	16.9	NA	33	[33]
*Scenedesmus obliquus*	Tubular PBR (0.06)	Taiwan, summer	NA	245.8	42.4	[38]
*Scenedesmus obliquus*	Raceway pond (4.5)	Portugal	1.19	12.6	29	[32]
*Chlorella vulgaris*	Thin-layer PBR (0.25)	Czech Republic	NA	1538.5	50	[39]
*Arthrospira platensis*	Raceway pond (0.3)	Israel	22.4	NA	NA	[35]
*Arthrospira platensis*	Raceway pond (0.3)	Israel, summer	24.8	NA	NA	[36]
*Arthrospira platensis*	Raceway pond (180)	IM, China, summer	27.5	100.8	46.4	This study

*P*_*ba*_, areal biomass productivity; *P*_*bv*_, volumetric biomass productivity; *C*_*c*_, carbohydrate content; IM, Inner Mongolia; NA, data not available

### Extraction and purification of water-soluble polysaccharide from *A. platensis*

#### Extraction of water-soluble polysaccharide from *A. platensis*

To extract and purify the water-soluble polysaccharide contained in the carbohydrate-enriched *A. platensis*, different extraction methods were compared with their efficiencies shown in Table 2. In the absence of cell disruption, the efficiency of direct hot water extraction (HWE) was only 38.75%. Similarly, Matloub et al. [41] reported an efficiency of 30–40% with direct hot or cold water extraction for the water-soluble polysaccharide from *A. platensis*. The extraction efficiency was relatively low probably due to the shielding effect of the algal cell wall which hindered the mass transfer of the extraction process. Therefore, the algae were subjected to physical disruption before water extraction. The most frequently used method for cell disruption is ultrasonication, where cavitation effect leads to destruction of cellular walls [21]. With the aid of ultrasonication, the extraction efficiency was improved by 18–117%. The best extraction efficiency of 84.09% was achieved under ultrasonication + HWE method with a solid–liquid ratio of 1:20. Oh et al. [42] also reported an improvement (at least 25–30%) of polysaccharide extraction yield from *Spirulina maxima* using ultrasonication-assisted hot water extraction method compared to the hot water extraction method alone. Increase of solid load (1:10, w/v) led to a dramatic decline of extraction efficiency, with almost only a half of that (45.87%) obtained under 1:20 (Table 2). Another physical disruption method applied herein was high-pressure homogenization, in which the cell suspension is forced to flow through a small orifice where turbulence, shear stress, and cavitation can promote cell lysis [43]. As shown in Table 2, the polysaccharide extraction efficiency reached up to 86.76% after the high-pressure homogenization + HWE treatment with a solid–liquid ratio of 1:30, which was 1.2-fold higher than the direct HWE and comparable to the result from the ultrasonication-assisted process. However, further increase of solid load (1:20, w/v) for high-pressure homogenization resulted in the clogged effect of the orifice.

**Table 2:** The extraction efficiency of *A. platensis* polysaccharide with different disruption technologies

Method (w/v)	Polysaccharide extraction efficiency (%)
Direct hot water extraction (HWE) (1:30)	38.75 ± 2.38
Ultrasonication + HWE (1:10)	45.87 ± 3.39
Ultrasonication + HWE (1:20)	84.09 ± 5.97
Ultrasonication + HWE (1:30)	79.64 ± 5.46
High-pressure homogenization + HWE (1:30)	86.76 ± 5.57

The present study demonstrated that the extraction of water-soluble polysaccharide from carbohydrate-enriched *A. platensis* could be facilitated with the assistance of physical destruction of algal cells. In fact, the cell wall of *A. platensis* is made up of four longitudinal layers with 10–15 nm thick for each layer composed mainly of murein, and the trichome was further enveloped by a thin mucilaginous sheath [12]. Therefore, these structures could become barriers for the water extraction of intracellular polysaccharide, which needed the aid of physical destruction. Ultrasonication and high-pressure homogenization were both ideal to assist the HWE process, with almost 1.2-fold improvement of extraction efficiency achieved herein in the carbohydrate-enriched *A. platensis* (Table 2). Although less water was required under the operation of ultrasonication (1:20, w/v) compared with high-pressure homogenization (1:30, w/v), the more convenience in industrially scaling up with high-pressure homogenization method due to its continuous operation made it more viable.

#### Purification of the extracted water-soluble polysaccharide from *A. platensis*

The polysaccharide purity of the crude extract was only 52.31%. To remove the protein and other impurities from the crude polysaccharide, different flocculation methods were applied. The efficiency of various types of flocculants, including polyferric sulfate, polyacrylamide, chitosan, and biological agents ZTC1 + 1, were assessed. As shown in Table 3, polyferric sulfate and ZTC1 + 1 could improve the polysaccharide purity by 33–59%, and the effects were positively dosage-dependent. The best polysaccharide purity of 83.37% was obtained under the application of ZTC1 + 1 with 1400 mg L^−1^ B and 700 mg L^−1^ A. Interestingly, the application of chitosan, which had been demonstrated to efficiently improve polysaccharide purity from 67 to 86.5% in the normal (protein-enriched) *A. platensis* [25], was inefficient in enhancing polysaccharide purity herein. As for the polysaccharide recovery, addition of ZTC1 + 1 also showed the highest polysaccharide retention rate of more than 81%, while other flocculation methods led to greater loss of polysaccharides, with chitosan displaying the lowest polysaccharide recovery (Table 3). Considering that threefold increase of ZTC1 + 1 dosage (350B + 175A to 1400B + 700A) could only slightly enhance polysaccharide purity (80.90% to 83.37%) while conversely decrease the polysaccharide retention rate (86.73% to 81.49%), it should be advisable to use ZTC1 + 1 with a dosage of 350B + 175A.

**Table 3:** The polysaccharide purity and recovery from *A. platensis* under different flocculant treatments

Method	Dosage (mg L^−1^)	Polysaccharide purity (%)	Polysaccharide retention rate (%)	Total polysaccharide recovery rate (%)^a^
Crude extract		52.31 ± 1.35	100	86.76 ± 5.57^a^
Polyferric sulfate	250	62.40 ± 2.56	58.49 ± 1.95	50.75 ± 1.78
	500	69.76 ± 2.67	63.01 ± 1.15	54.67 ± 1.94
	750	76.80 ± 3.35	68.42 ± 0.97	59.36 ± 1.69
Polyacrylamide 150	200	53.97 ± 1.27	78.07 ± 2.01	67.73 ± 1.98
Polyacrylamide 170	200	56.31 ± 1.31	79.78 ± 1.57	69.22 ± 1.79
Chitosan	600	60.73 ± 1.36	57.83 ± 1.09	50.17 ± 0.99
	900	59.25 ± 1.29	56.81 ± 1.21	49.29 ± 0.86
	1200	56.67 ± 1.29	55.01 ± 0.52	47.73 ± 0.59
ZTC1 + 1	350B + 175A	80.90 ± 1.01	86.73 ± 1.98	75.25 ± 1.31
	700B + 350A	82.05 ± 0.21	81.14 ± 2.03	70.40 ± 0.66
	1400B + 700A	83.37 ± 0.14	81.15 ± 2.24	70.70 ± 0.54
ZTC1 + 1 + Sevage (two times)	350B + 175A	98.90 ± 1.01	78.67 ± 2.11	68.25 ± 1.13

^a^The total polysaccharide recovery was assessed based on the high-pressure homogenization + HWE process

ZTC1 + 1 is a commercialized non-toxic natural clarifying agent which comprises two components, one of which acts as the main flocculants and the other as an auxiliary. Although the mechanism of this agent for clarification is unclear hitherto, it has been widely used in the clarification of the decoction of traditional Chinese medicine due to its high efficiency in removing protein, tannins and gums while retaining polysaccharides with safety [44]. To the best of our knowledge, the present study was the first trial on the application of ZTC1 + 1 for the purification of polysaccharide from alga *A. platensis*. Compared with other flocculation methods, ZTC1 + 1 resulted in not only the highest polysaccharide purity, but also the least polysaccharide loss. The polysaccharide purity after ZTC1 + 1 treatment (> 80%) was even 22% higher than that with enzymatic trichloroacetic acid (TCA) method where 3% papain and 5% TCA were sequentially used to remove impurities in the crude polysaccharide extracts from *A. platensis* [24]. Compared with traditional deproteinization methods such as Sevage and TCA method where organic reagents and complicated purification process are often involved (e.g., 5–10 times repeated treatments in Sevage method) [23], ZTC1 + 1 is an effective, simple and environmental-friendly way for polysaccharide purification as an impurity scavenger from algae and other natural products. Considering the extraction efficiency in the high-pressure homogenization + HWE process, the total polysaccharide recovery rate reached 75.25% with a polysaccharide purity of 80.90% using 350B + 175A of ZTC1 + 1 (Table 3).

To characterize the structure of the polysaccharide, Sevage method was applied to further refine the polysaccharide following the ZTC1 + 1 method. After the Sevage treatment for two times, the polysaccharide purity reached 98.9% (Table 3), which was even slightly higher than that obtained with chitosan flocculation-macroporous resin adsorption (96.2%) [25].

### Characterization of water-soluble polysaccharide from *A. platensis*

**Fig. 3:**
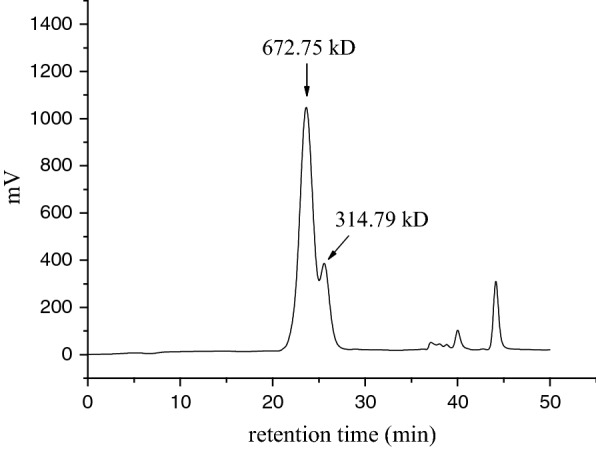
Size exclusion chromatography of polysaccharides from nitrogen-limited *A. platensis*

Size exclusion chromatography analysis showed that two populations, with molecular weight of 673 kDa and 315 kDa, could be discerned, although they were not completely separated (Fig. 3). The size of the polysaccharides characterized herein fell well into the known *A. platensis* polysaccharides reported in the literatures (Table 4).

**Table 4:** Molecular weight, glycosidic linkage, and monosaccharide composition of polysaccharides from different *A. platensis*

Polysaccharide	Molecular weight (kDa)	Configuration of glycosides	Glycosidic linkage	Monosaccharide composition (%)									References
				Glc	Rha	Ara	Xyl	Man	Gal	Fuc	GlcUA	GalUA	
PS-NL^a^	673 and 315	α	1,3 and 1,2 or 1,4	91.41	0.93	0.46	0.32	0.36	0.34	0.40	0	0.59	This study
PSP-3c	12.33	α	–^b^	> 90	Minor	–	–	Minor	–	–	–	–	[23]
PS	500 and 70	–	1,4	42.5	–	–	–	57.5	–	–	–	–	[20]
PS	1400, 420, and 2	–	–	90.87	2.25	0.68	0.92	1.68	2.40	0.99	–	–	[25]
Glucan	–	α	1,4 (major) and 1,6 (minor)	98.2	–	–	–	–	–	–	–	–	[49]
SPPA-1	690	α	1,4 (major) and 1,6 (minor)	100	–	–	–	–	–	–	–	–	[50]
PUF2	165	–	–	4.3	49.7	–	5.9	0.9	5.8	–	15.1	16.9	[45]
PSP	–	–	–	21.3	43.6	1.1	2.4	–	1.3	–	–	–	[24]
Immulina polysaccharide	> 1000	–	1,3 and 1,4	3.6	35.4	1.8	5.5	2.4	7.1	7.7	9.7	2.0	[46]
Ca-SP	–	–	1,3 and 1,2 or 1,4	–	52.3	–	–	–	–	–	–	–	[47]

^a^Polysaccharide derived from nitrogen-limited *A. platensis*

^b^Data unavailable

The monosaccharide composition of the purified polysaccharide from nitrogen-limited *A. platensis* (PS-NL) was analyzed by gas chromatography (Additional file 1: Fig. S2). Table 4 showed that PS-NL was predominantly composed of glucose (Glc), which accounted for more than 91% of the total sugar. Very few of other monosaccharides and derivatives could be detected, including rhamnose (Rha), arabinose (Ara), fucose (Fuc), mannose (Man), galactose (Gal), xylose (Xyl) and galacturonic acid (GalUA) with each component accounting for less than 1%. Similarly, Wu et al. [23] and Wang et al. [25] also reported that glucose was the main component (> 90%) of the polysaccharides from *A. platensis* (Table 4). However, considerable amounts of other studies revealed that instead of Glc, Rha accounted for the main sugar moiety of the polysaccharides from *A. platensis* (Table 4), with the proportion of which reaching up to approximately 50% [24, 45–47]. Additionally, Chen et al. [20] found that *A. platensis* water-soluble polysaccharides were composed of 57.5% Man and 42.5% Glc, which was quite different from the results discussed above.

Infrared spectrum (Additional file 1: Fig. S3) of PS-NL within the range of 4000–400 cm^−1^ displayed the characteristic features of polysaccharide: a strong OH stretch at 3200–3500 cm^−1^ and a strong OH bend at 1642 cm^−1^. Absorbance bands at 2930 cm^−1^ corresponded to C–H in the glycosidic cycles. Thin and strong absorbance bands at 1023–1154 cm^−1^ represented vibrations of C–O–C, C–OH and C–C ring in the glycosidic cycles. The occurrence of uronic acids was suggested by the absorbance band at 1414 cm^−1^ (O–C=O bending). Absorption at 850 cm^−1^ rather than at 890 cm^−1^ suggested an α-type instead of β-type glycosidic linkage of the polysaccharide [29]. ^1^H NMR spectrum (Additional file 1: Fig. S4a) showed peaks from 3.4 to 4.0 corresponding to C–H in the glycosidic residue. Specifically, a strong peak at 5.4 ppm was detected, which could be assigned as an α-type anomeric carbon [29]. There was no peak at 4.4–4.7 ppm, indicating that β-type glycoside was absent from the polysaccharide [29]. The strong peak at 101 ppm in the ^13^C NMR spectrum (Additional file 1: Fig. S4b) also indicated the presence of an α-type anomeric carbon, whereas the absence of peaks at 103–106 ppm suggested no β-type isoforms [29], which was in agreement with the results from IR and ^1^H NMR. The peak at 61.8 ppm represented the C-6 of the glycosidic residue, while peaks of 70.6–78.2 were corresponded to C2–C5. There was no peak between 170 and 180 ppm, indicating that no modification, such as acetylation [48], was present in the polysaccharide. Collectively, IR and NMR analysis demonstrated that PS-NL contained α-type glycosidic linkages, which was consistent with the glycosidic configuration of the majority of the reported polysaccharides from *A. platensis* (Table 4).

Periodic acid oxidation analysis showed that 0.49 mol of periodate was consumed per mole of hexose residue with 0.013 mol of formic acid produced, indicating that the polysaccharide mainly contained 1→3 and 1→4 or 1→2 glycosidic linkage with very small proportion of 1→6 glycosidic linkage; the rate of glycosidic linkage of 1→3, 1→4 or 1→2, and 1→6 was calculated to be about 46.7:52:1.3 according to [29]. 1→4 and 1→2 glycosidic linkage cannot be distinguished with periodic acid oxidation analysis, which needs more comprehensive characterization. Collectively, PS-NL was demonstrated to be dominantly composed of (1→3)(1→4) or (1→3)(1→2)-α-glucan. Although (1→3)(1→4) or (1→3)(1→2) linkage had been found in several polysaccharides of *A. platensis*, the main monosaccharide moiety was Rha instead of Glc [46, 47]. In contrast, Sekharam et al. [49] and Wang et al. [50] reported two kinds of α-glucans from *A. platensis*, both of which, however, contained glycosidic linkages of 1→4 (major) and 1→6 (minor). Compared with the known polysaccharide of *A. platensis* (Table 4), the structure of PS-NL herein was unique. It should be noted that the polysaccharides reported in the literatures were extracted from normal *A. platensis* which was grown under favorable conditions with a carbohydrate content generally less than 15% [12], whereas PS-NL in the present study originated from nitrogen-limited *A. platensis* in which carbohydrate content reached more than 60%. It is acknowledged that under nitrogen limitation, *A. platensis* accumulates storage polysaccharide with the primary form of glycogen, which is composed of monomeric glucose units with highly branched (1→4)(1→6)-linked α-glucan [3, 14, 17]. However, interestingly, the present study exhibited a new type of polysaccharide derived from nitrogen-limited *A. platensis* which was distinct from the commonly known glycogen or starch-type storage α-glucan. Iodine-staining analysis also revealed the unique feature of PS-NL compared with the α-glucan such as glycogen, amylose, amylopectin, and dextran (Additional file 1: Fig. S5).

In general, the polysaccharides of cyanobacteria include storage polysaccharides (SPS) that are accumulated intracellularly, extracellular polysaccharides (EPS) or released polysaccharides (RPS) that release in the culture medium, and capsular polysaccharides (CPS) or bound polysaccharides (BPS) that are associated with cell wall [51–53]. Under nitrogen limitation condition, the main carbohydrate in *A. platensis* was SPS (formerly considered as glycogen), usually accounting for 40–65%DW [51]. The CPS or BPS, which were usually extracted by hot water, accounted for less than 8%DW under nitrogen limitation and their contents were negatively related to nitrogen supply [54]. The EPS or RPS should have been removed from the algal biomass by filtration and water wash. Collectively, it was reasonable to conclude that the final water-soluble polysaccharide obtained in the present study was dominated by the SPS, with very little RPS or CPS involved. Therefore, it was clear that the storage polysaccharide in *A. platensis*, as characterized herein, was a (1a3)(1→4)- or (1→3)(1→2)-α-glucan instead of commonly acknowledged glycogen. (1→3)(1→4)-α-glucan can generally be found in fungi (e.g., *Aspergillus niger*) and lichens (e.g., *Cetraria islandica*) [55]. The presence of this type of polysaccharide in nitrogen-limited *A. platensis* was rarely reported, which needed more effort to dissect its biosynthesis and the underlying regulatory mechanism in response to environment.

The present study revealed the possibility of industrially producing α-glucan-enriched *A. platensis* under photoautotrophic conditions in outdoor open raceway ponds. If semi-continuous cultivation mode was applied, the production process should have excellent compatibility with the existing *A. platensis*-producing systems with nearly no additional or even reduced cost involved because of the less nitrogen fertilizer supplied. The biomass productivity should also be attractive, as discussed above. The current average annual productivity of protein-enriched *A. platensis* in Ordos is estimated to be 12 t ha^−1^ year^−1^ [26], which is twofold of that of grains such as wheat and corn, and threefold of tuber crops in China in 2018 (http://www.stats.gov.cn/tjsj/zxfb/201812/t20181214_1639544.html). Given that the production of α-glucan-enriched *A. platensis* had the comparable productivity with the protein-enriched one, and the high-value-added components such as protein (more than 35%DW remained, Fig. 2e) were incorporated into the industrial chain for additional products (e.g., aquafeed additives or active peptides), it would be possible to partially replace corn as the glucose source by this α-glucan-enriched alga in the field of chemical and fermentation industry. This will alleviate the demand for agricultural crops (e.g., corn) as the feedstock for biorefinery. In addition, the potential application of the α-glucan itself from *A. platensis* should be further developed. In fact, the structure of this α-glucan resembled that of the immunologically active (1→3)(1→4)-α-glucan from *Cetraria islandica* [56], which suggested a possible activity in immunoregulation. These high-value applications could contribute to the economic viability of the carbohydrate-based biorefinery from *A. platensis*.

## Conclusions

The feasibility of producing carbohydrate-enriched *A. platensis* in industrial-scale outdoor raceway ponds was demonstrated, with a maximum biomass productivity of 27.5 g m^−2^ day^−1^ and the corresponding carbohydrate content of 46.5%DW obtained under nitrogen limitation. Efficient extraction of the water-soluble polysaccharide from this alga relied on high-pressure homogenization in addition to hot water extraction. ZTC1 + 1 flocculation was an effective way for polysaccharide purification from *A. platensis*. PS-NL from nitrogen-limited *A. platensis* was unique to be composed of (1→3) (1→4)- or (1→3) (1→2)-α-glucan. The α-glucan from *A. platensis* could be a potential glucose source for industrial applications.

## Additional file

**Additional file 1: Figure S1.** Temperature variations in the culture of *A. platensis* in industrial-scale open raceway ponds under nitrogen-limited conditions. **Figure S2.** Gas chromatography analysis of monosaccharide composition of polysaccharide from nitrogen-limited *A. platensis*. **Figure S3.** FTIR analysis of polysaccharide from nitrogen-limited *A. platensis*. **Figure S4.** ^1^H NMR and ^13^C NMR spectrum of polysaccharide from nitrogen-limited *A. platensis.* **Figure S5.** Iodine-staining analysis of different polysaccharides and comparison with the polysaccharide from nitrogen-limited *A. platensis* (PS-NL).

## Data Availability

All data generated or analyzed during this study are included in this published article and Additional file 1.

## References

[1] Nigam PS, Singh A (2011). Production of liquid biofuels from renewable resources. Prog Energy Combust Sci.

[2] Zhou W, Li Y, Min M, Hu B, Chen P, Ruan R (2011). Local bioprospecting for high-lipid producing microalgal strains to be grown on concentrated municipal wastewater for biofuel production. Bioresour Technol.

[3] Aikawa S, Ho SH, Nakanishi A, Chang JS, Hasunuma T, Kondo A (2015). Improving polyglucan production in cyanobacteria and microalgae via cultivation design and metabolic engineering. Biotechnol J.

[4] Dismukes GC, Carrieri D, Bennette N, Ananyev GM, Posewitz MC (2008). Aquatic phototrophs: efficient alternatives to land-based crops for biofuels. Curr Opin Biotechnol.

[5] Chen C-Y, Zhao X-Q, Yen H-W, Ho S-H, Cheng C-L, Lee D-J, Bai F-W, Chang J-S (2013). Microalgae-based carbohydrates for biofuel production. Biochem Eng J.

[6] John RP, Anisha GS, Nampoothiri KM, Pandey A (2011). Micro and macroalgal biomass: a renewable source for bioethanol. Bioresour Technol.

[7] Mathiot C, Ponge P, Gallard B, Sassi J-F, Delrue F, Le Moigne N (2019). Microalgae starch-based bioplastics: screening of ten strains and plasticization of unfractionated microalgae by extrusion. Carbohydr Polym.

[8] Yen HW, Hu IC, Chen CY, Ho SH, Lee DJ, Chang JS (2013). Microalgae-based biorefinery—from biofuels to natural products. Bioresour Technol.

[9] Yao C, Jiang J, Cao X, Liu Y, Xue S, Zhang Y (2018). Phosphorus enhances photosynthetic storage starch production in a green microalga (Chlorophyta) *Tetraselmis subcordiformis* in nitrogen starvation conditions. J Agric Food Chem.

[10] Braga VDS, Mastrantonio D, Costa JAV, Morais MG (2018). Cultivation strategy to stimulate high carbohydrate content in *Spirulina* biomass. Bioresour Technol.

[11] Chen J, Wang Y, Benemann JR, Zhang X, Hu H, Qin S (2016). Microalgal industry in China: challenges and prospects. J Appl Phycol.

[12] Belay A, Richmond A, Hu Q (2013). Biology and industrial production of *Arthrospira* (*Spirulina*). Handbook of microalgal culture: applied phycology and biotechnology.

[13] Hasunuma T, Kikuyama F, Matsuda M, Aikawa S, Izumi Y, Kondo A (2013). Dynamic metabolic profiling of cyanobacterial glycogen biosynthesis under conditions of nitrate depletion. J Exp Bot.

[14] Aikawa S, Izumi Y, Matsuda F, Hasunuma T, Chang JS, Kondo A (2012). Synergistic enhancement of glycogen production in *Arthrospira platensis* by optimization of light intensity and nitrate supply. Bioresour Technol.

[15] Yao C, Pan Y, Lu H, Wu P, Meng Y, Cao X, Xue S (2016). Utilization of recovered nitrogen from hydrothermal carbonization process by *Arthrospira platensis*. Bioresour Technol.

[16] Markou G, Angelidaki I, Nerantzis E, Georgakakis D (2013). Bioethanol production by carbohydrate-enriched biomass of *Arthrospira* (*Spirulina*) *platensis*. Energies..

[17] Izumi Y, Aikawa S, Matsuda F, Hasunuma T, Kondo A (2013). Aqueous size-exclusion chromatographic method for the quantification of cyanobacterial native glycogen. J Chromatogr B Anal Technol Biomed Life Sci..

[18] Ball SG, Morell MK (2003). From bacterial glycogen to starch: understanding the biogenesis of the plant starch granule. Annu Rev Plant Biol.

[19] Chaiklahan R, Chirasuwan N, Triratana P, Loha V, Tia S, Bunnag B (2013). Polysaccharide extraction from *Spirulina* sp. and its antioxidant capacity. Int J Biol Macromol..

[20] Chen HW, Yang TS, Chen MJ, Chang YC, Lin CY, Wang EI, Ho CL, Huang KM, Yu CC, Yang FL (2012). Application of power plant flue gas in a photobioreactor to grow *Spirulina* algae, and a bioactivity analysis of the algal water-soluble polysaccharides. Bioresour Technol.

[21] Kurd F, Samavati V (2015). Water soluble polysaccharides from *Spirulina platensis*: extraction and in vitro anti-cancer activity. Int J Biol Macromol.

[22] Silva AS, de Magalhães WT, Moreira LM, Rocha MVP, Bastos AKP (2018). Microwave-assisted extraction of polysaccharides from *Arthrospira* (*Spirulina*) *platensis* using the concept of green chemistry. Algal Res..

[23] Wu X, Li R, Zhao Y, Liu Y (2017). Separation of polysaccharides from *Spirulina platensis* by HSCCC with ethanol-ammonium sulfate ATPS and their antioxidant activities. Carbohydr Polym.

[24] Wang B, Liu Q, Huang Y, Yuan Y, Ma Q, Du M, Cai T, Cai Y (2018). Extraction of polysaccharide from *Spirulina* and evaluation of its activities. Evid Based Complement Alternat Med..

[25] Wang F, Ma Y, Liu Y, Cui Z, Ying X, Zhang F, Linhardt RJ (2017). A simple strategy for the separation and purification of water-soluble polysaccharides from the fresh *Spirulina platensis*. Sep Sci Technol.

[26] Lu YM, Xiang WZ, Wen YH (2011). S*pirulina (Arthrospira)* industry in inner Mongolia of China: current status and prospects. J Appl Phycol.

[27] Dubois M, Gilles KA, Hamilton JK, Rebers PA, Smith F (1956). Colorimetric method for determination of sugars and related substances. Anal Chem.

[28] Alsop RM, Vlachogiannis GJ (1982). Determination of the molecular weight of clinical dextran by gel permeation chromatography on TSK PW type columns. J Chromatogr.

[29] Jahanbin K (2018). Structural characterization of a new water-soluble polysaccharide isolated from *Acanthophyllum acerosum* roots and its antioxidant activity. Int J Biol Macromol.

[30] Kim CJ, Jung YH, Oh HM (2007). Factors indicating culture status during cultivation of *Spirulina* (*Arthrospira*) *platensis*. J Microbiol..

[31] Hidasi N, Belay A (2018). Diurnal variation of various culture and biochemical parameters of *Arthrospira platensis* in large-scale outdoor raceway ponds. Algal Res.

[32] Miranda JR, Passarinho PC, Gouveia L (2012). Bioethanol production from *Scenedesmus obliquus* sugars: the influence of photobioreactors and culture conditions on biomass production. Appl Microbiol Biotechnol.

[33] Wen X, Du K, Wang Z, Peng X, Luo L, Tao H, Xu Y, Zhang D, Geng Y, Li Y (2016). Effective cultivation of microalgae for biofuel production: a pilot-scale evaluation of a novel oleaginous microalga *Graesiella* sp. WBG-1. Biotechnol Biofuels..

[34] Eustance E, Wray JT, Badvipour S, Sommerfeld MR (2015). The effects of cultivation depth, areal density, and nutrient level on lipid accumulation of *Scenedesmus acutus* in outdoor raceway ponds. J Appl Phycol.

[35] Vonshak A, Laorawat S, Bunnag B, Tanticharoen M (2013). The effect of light availability on the photosynthetic activity and productivity of outdoor cultures of *Arthrospira platensis* (*Spirulina*). J Appl Phycol.

[36] Richmond A, Lichtenberg E, Stahl B, Vonshak A (1990). Quantitative assessment of the major limitations on productivity of *Spirulina platensis* in open raceways. J Appl Phycol.

[37] He Q, Yang H, Hu C (2016). Culture modes and financial evaluation of two oleaginous microalgae for biodiesel production in desert area with open raceway pond. Bioresour Technol.

[38] Ho SH, Chen YD, Chang CY, Lai YY, Chen CY, Kondo A, Ren NQ, Chang JS (2017). Feasibility of CO2 mitigation and carbohydrate production by microalga *Scenedesmus obliquus* CNW-N used for bioethanol fermentation under outdoor conditions: effects of seasonal changes. Biotechnol Biofuels.

[39] Branyikova I, Marsalkova B, Doucha J, Branyik T, Bisova K, Zachleder V, Vitova M (2011). Microalgae–novel highly efficient starch producers. Biotechnol Bioeng.

[40] Yang H, He Q, Hu C (2018). Feasibility of biodiesel production and CO_2_ emission reduction by *Monoraphidium dybowskii* LB50 under semi-continuous culture with open raceway ponds in the desert area. Biotechnol Biofuels.

[41] Matloub AA, El-Senousy WM, Elsayed AB, ElSouda S, Aly H (2013). Anti-HCV, antioxidant, cytotoxic and hypolipidemic activities of water soluble polysaccharides of *Spirulina platensis*. Planta Med..

[42] Oh S-H, Ahn J, Kang D-H, Lee H-Y (2011). The effect of ultrasonificated extracts of *Spirulina maxima* on the anticancer activity. Mar Biotechnol.

[43] Diels AMJ, Michiels CW (2006). High-pressure homogenization as a non-thermal technique for the inactivation of microorganisms. Crit Rev Microbiol.

[44] Zhang P, Wu Y, Hua L (2007). Clarifying effect of ZTC1 + 1-IIclarifyicant for decoction of traditional Chinese medicine. China J Chin Mater Med..

[45] Majdoub H, Ben Mansour M, Chaubet F, Roudesli MS, Maaroufi RM (2009). Anticoagulant activity of a sulfated polysaccharide from the green alga *Arthrospira platensis*. Biochim Biophys Acta.

[46] Pugh N, Ross SA, Elsohly HN, Elsohly MA, Pasco DS (2001). Isolation of three high molecular weight polysaccharide preparations with potent immunostimulatory activity from *Spirulina platensis*, aphanizomenon flos-aquae and *Chlorella pyrenoidosa*. Planta Med.

[47] Lee J, Hayashi TK, Sankawa U, Maeda M, Nemoto T, Nakanishi H (1998). Further purification and structural analysis of calcium spirulan from *Spirulina platensis*. J Nat Prod..

[48] Ravenscroft N, Cescutti P, Gavini M, Stefanetti G, MacLennan CA, Martin LB, Micoli F (2015). Structural analysis of the *O*-acetylated *O*-polysaccharide isolated from *Salmonella paratyphi* A and used for vaccine preparation. Carbohydr Res.

[49] Sekharam KM, Venkataraman LV, Salimath PV (1989). Structural studies of a glucan isolated from blue-green alga *Spirulina platensis*. Food Chem.

[50] Wang Z, Peng X, Huang L, Peng Z, Tian G (2001). Structure elucidation of glycan of a glycoconjugate SPPA-1 isolated from *Spirulina platensis*. Acta Pharm Sin..

[51] Phélippé M, Gonçalves O, Thouand G, Cogne G, Laroche C (2019). Characterization of the polysaccharides chemical diversity of the cyanobacteria *Arthrospira platensis*. Algal Res.

[52] Bernaerts TMM, Gheysen L, Kyomugasho C, Jamsazzadeh Kermani Z, Vandionant S, Foubert I, Hendrickx ME, Van Loey AM (2018). Comparison of microalgal biomasses as functional food ingredients: focus on the composition of cell wall related polysaccharides. Algal Res.

[53] De Philippis R, Sili C, Paperi R, Vincenzini M (2001). Exopolysaccharide-producing cyanobacteria and their possible exploitation: a review. J Appl Phycol.

[54] Elbaky HA, Elbaz KFH, Ellatife SA (2014). Induction of sulfated polysaccharides in *Spirulina platensis* as response to nitrogen concentration and its biological evaluation. J Aquacult Res Dev.

[55] Synytsya A, Novak M (2014). Structural analysis of glucans. Ann Transl Med..

[56] Olafsdottir ES, Ingolfsdottir K, Barsett H, Smestad Paulsen B, Jurcic K, Wagner H (1999). Immunologically active (1→3)-(1→4)-α-d-glucan from *Cetraria islandica*. Phytomedicine.

