# Supplementary material for: Production and structural characterization of a new type of polysaccharide from nitrogen-limited Arthrospira platensis cultivated in outdoor industrial-scale open raceway ponds
Source: Biotechnol Biofuels. 2019 May 24;12:131. doi: 10.1186/s13068-019-1470-3 (PMC6533678; doi:10.1186/s13068-019-1470-3)
Supplement: Supplementary file 1 — Additional file 1: Figure S1. Temperature variations in the culture of A. platensis in industrial-scale open raceway ponds under nitrogen-limited conditions. Figure S2. Gas chromatography analysis of monosaccharide composition of polysaccharide from nitrogen-limited A. platensis. Figure S3. FTIR analysis of polysaccharide from nitrogen-limited A. platensis. Figure S4. 1H NMR and 13C NMR spectrum of polysaccharide from nitrogen-limited A. platensis. Figure S5. Iodine-staining analysis of different polysaccharides and comparison with the polysaccharide from nitrogen-limited A. platensis (PS-NL). [file 13068_2019_1470_MOESM1_ESM.docx]

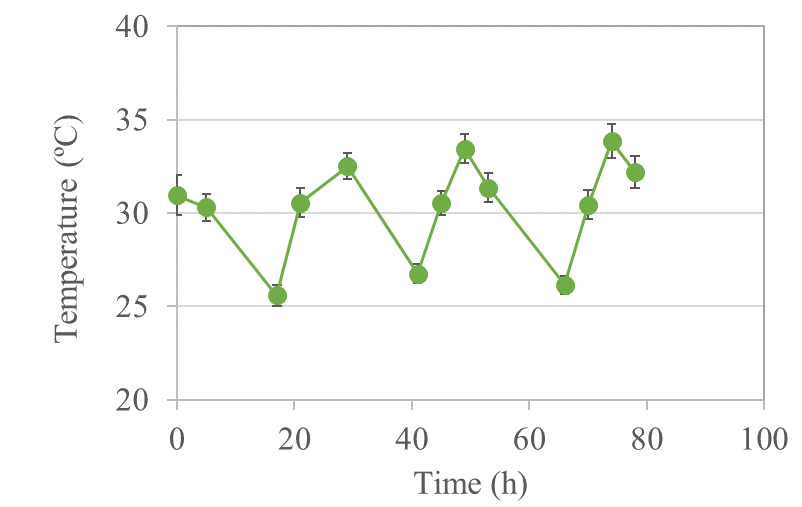


**Fig. S1.** Temperature variations in the culture of *A. platensis* in industrial-scale open raceway ponds under nitrogen-limited conditions.

**Fig. S2.** Gas chromatography analysis of monosaccharide composition of polysaccharide from nitrogen-limited *A. platensis.* a, rhamnose; b, fucose; c, arabinose; d, xylose; e, mannose; f, galactose; g, glucose; h, glucuronic acid; I, galacturonic acid.

**Fig. S3.** FTIR analysis of polysaccharide from nitrogen-limited *A. platensis.*


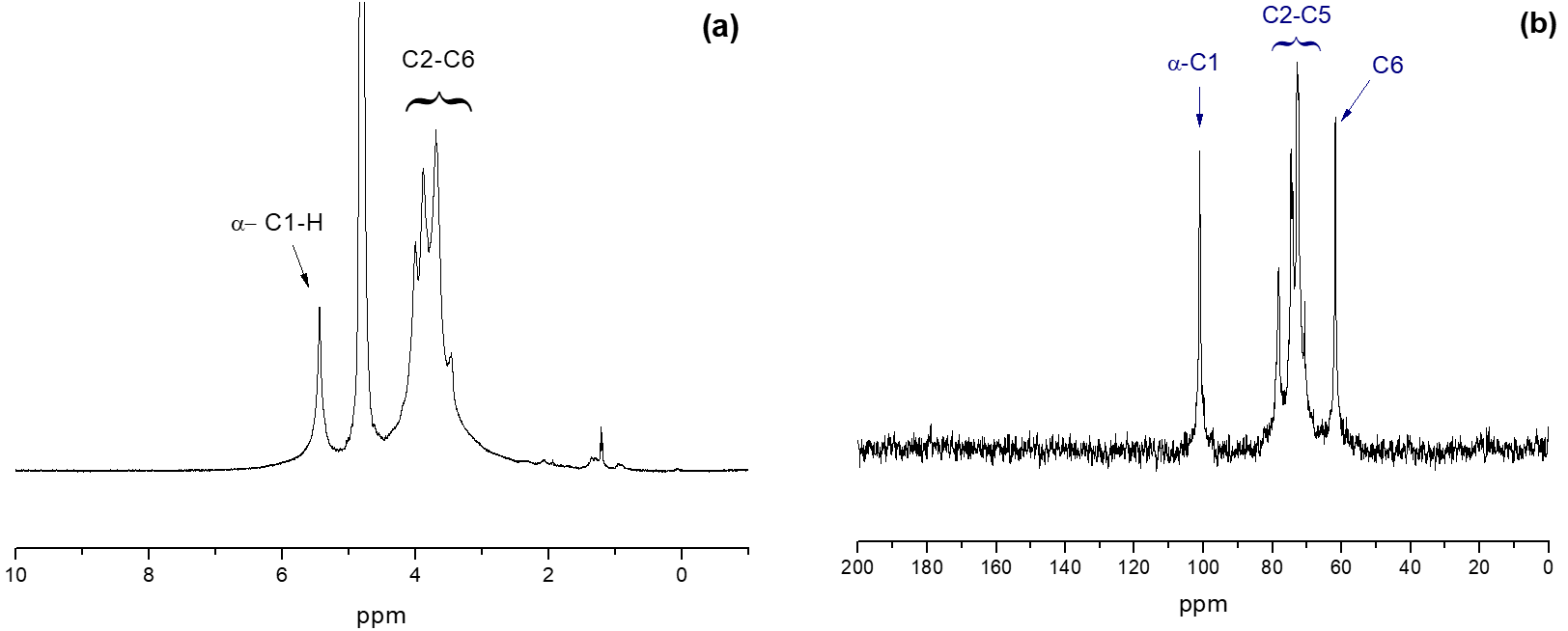


**Fig. S4.** ^1^H NMR and ^13^C NMR spectrum of polysaccharide from nitrogen-limited *A. platensis.*


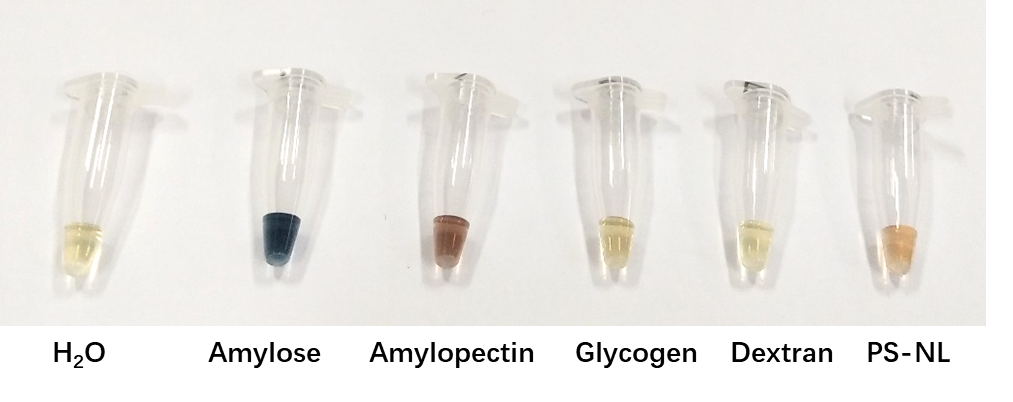


**Fig. S5.** Iodine-staining analysis of different polysaccharides and comparison with the polysaccharide from nitrogen-limited *A. platensis* (PS-NL)*.* 20 μL 0.25 g/L glucan+4 μL 0.01N I_2_-KI solution.
